# Leptin and inflammatory factors play a synergistic role in the regulation of reproduction in male mice through hypothalamic kisspeptin-mediated energy balance

**DOI:** 10.1186/s12958-021-00698-0

**Published:** 2021-01-20

**Authors:** Bo Chang, Chenglin Song, Haining Gao, Tie Ma, Tao Li, Qianhe Ma, Tingting Yao, Meng Wang, Jing Li, Xuejie Yi, Donghui Tang, Shicheng Cao

**Affiliations:** 1 Exercise and Health Research Center/Department of Kinesiology, Shenyang Sport University, Shenyang, 110102 Liaoning China; 2 PE College of Beijing Normal University, Beijing, 100875 China; 3 Department of Sports Medicine, School of Public and Basic Sciences, China Medical University, Shenyang, 110122 Liaoning China

**Keywords:** Energy balance, Inflammation, Leptin, Kisspeptin, Hypogonadotropic hypogonadism

## Abstract

**Background:**

Energy balance is closely related to reproductive function, wherein hypothalamic kisspeptin mediates regulation of the energy balance. However, the central mechanism of kisspeptin in the regulation of male reproductive function under different energy balance states is unclear. Here, high-fat diet (HFD) and exercise were used to change the energy balance to explore the role of leptin and inflammation in the regulation of kisspeptin and the hypothalamic-pituitary-testis (HPT) axis.

**Methods:**

Four-week-old male C57BL/6 J mice were randomly assigned to a normal control group (*n* = 16) or an HFD (*n* = 49) group. After 10 weeks of HFD feeding, obese mice were randomly divided into obesity control (*n* = 16), obesity moderate-load exercise (*n* = 16), or obesity high-load exercise (*n* = 17) groups. The obesity moderate-load exercise and obesity high-load exercise groups performed exercise (swimming) for 120 min/day and 120 min × 2 times/day (6 h interval), 5 days/week for 8 weeks, respectively.

**Results:**

Compared to the mice in the normal group, in obese mice, the mRNA and protein expression of the leptin receptor, kiss, interleukin-10 (IL-10), and gonadotropin-releasing hormone (GnRH) decreased in the hypothalamus; serum luteinizing hormone (LH), follicle-stimulating hormone (FSH), and testosterone levels and sperm quality decreased; and serum leptin, estradiol, and tumor necrosis factor-α (TNF-α) levels and sperm apoptosis increased. Moderate- and high-load exercise effectively reduced body fat and serum leptin levels but had the opposite effects on the hypothalamus and serum IL-10 and TNF-α levels. Moderate-load exercise had anti-inflammatory effects accompanied by increased mRNA and protein expression of kiss and GnRH in the hypothalamus and increased serum FSH, LH, and testosterone levels and improved sperm quality. High-load exercise also promoted inflammation, with no significant effect on the mRNA and protein expression of kiss and GnRH in the hypothalamus, serum sex hormone level, or sperm quality. Moderate-load exercise improved leptin resistance and inflammation and reduced the inhibition of kisspeptin and the HPT axis in obese mice. The inflammatory response induced by high-load exercise may counteract the positive effect of improving leptin resistance on kisspeptin and HPT.

**Conclusion:**

During changes in energy balance, leptin and inflammation jointly regulate kisspeptin expression on the HPT axis.

## Background

The energy balance is closely related to reproductive function. Long-term energy imbalance affects the development and physiological function of the hypothalamic–pituitary–gonadal (HPG) axis in adolescence and adulthood [1, 2]. Both a negative energy balance caused by long-term food restriction [2] and positive energy balance induced by dietary obesity [3] can lead to decreased serum testosterone levels, spermatogenesis, and sexual dysfunction in men, often accompanied by a decrease in serum follicle-stimulating hormone (FSH) and luteinizing hormone (LH). This series of symptoms is known as hypogonadotropic hypogonadism (HH) [4].

Exercise is an important means for adjusting the energy balance. Several studies have confirmed that long-term moderate- or heavy-load exercise can effectively increase energy consumption, cause the body to have a negative energy balance, and reduce body fat. However, the effect of exercise on reproductive function remains controversial, as exercise has been reported to increase [5, 6], not affect [7], and decrease [8] serum testosterone levels and sperm quality. Our previous study showed that moderate- and heavy-load exercise increased energy consumption and reduced body fat in obese mice. However, only moderate-load exercise significantly improved gonadal hypofunction in obese male mice, while high-load exercise showed no significant effect [9]. The mechanism underlying this phenomenon was not clear. Recent studies confirmed that kisspeptin is vital for the neuroendocrine regulation of gonadotropin-releasing hormone (GnRH) secretion [10]. Therefore, we predicted that high-fat diet (HFD) and exercise-induced changes in the energy balance affect male reproductive function through hypothalamic kisspeptin.

Kisspeptin is a hypothalamic neuropeptide. Its receptor is expressed on all GnRH neurons. In vivo [11] and in vitro [12] studies have shown that kisspeptin or kiss agonists can activate GnRH neurons and induce the pituitary to release gonadotropin [13]. The stimulation of kiss is blocked by GnRH antagonists [14]. Kiss neurons are highly sensitive to the energy balance [10]. After 48 h of fasting in adult male mice, the expression of kiss and kiss receptor mRNA in the hypothalamus was decreased significantly [15]. In the hypothalamus [16] of obese mice, the expression of kiss and number of kisspeptin neurons were found to be decreased. However, the mechanism of kisspeptin-mediated regulation of energy balance in reproductive function is unclear. Some studies showed that kiss neurons express the leptin receptor, a metabolic regulator. Furthermore, leptin resistance caused by long-term intake of an HFD decreased the expression of OB Rb and kisspeptin in the mouse hypothalamus, affecting sexual development and leading to reproductive dysfunction in males [16]. Recent data showed that the kisspeptin system is sensitive to inflammation. Systemic injection of lipopolysaccharide (LPS) in female rats reduced the expression of *KiSS1* mRNA in the hypothalamus and inhibited the release of LH [17]. Intravenous injection of kisspeptin reversed LPS-induced LH inhibition [18]. In vitro, tumor necrosis factor-alpha (TNF-α) and interleukin-1 (IL-1) decreased GnRH secretion by downregulating kisspeptin signaling in the hypothalamus [19, 20]. Moreover, a long-term positive energy balance can lead to the development of leptin resistance and chronic inflammatory response, both of which are closely related to exercise [9, 21]. However, whether the two can work together to regulate the energy balance of the hypothalamic kiss neurons and regulate male reproductive function is unknown.

Therefore, in this study, we examined leptin and inflammatory factors in the blood and hypothalamus of obese mice (positive energy balance) and explored whether obesity can inhibit the function of the HPG axis by downregulating the expression of kisspeptin via leptin resistance and the inflammatory response. Analysis of obese mice that performed moderate- and heavy-load exercise (negative energy balance) was conducted to further determine the roles of hypothalamic leptin and inflammatory factors in kisspeptin-mediated energy balance regulation of reproduction and their relationship with exercise load. Our results provide insight into the role of physical activity in preventing obesity-related male infertility.

## Methods

### Animals

Seventy healthy male C57BL/6 mice (4–5 weeks old), weighing 17.36 ± 1.51 g, were provided by Beijing Weitong Lihua Experimental Animal Technology Co., Ltd. [license number SCXK (Jing) 2018–0008]. Animal experiments were performed in accordance with the Procedures for the Management and Use of Laboratory Animals issued by the Ministry of Health of the People’s Republic of China in 1998. All experimental studies were approved by the Ethics Committee of the Shenyang Institute of Physical Education. The animals were kept at a relative humidity of 50–60% and at 30–33 °C room temperature under a 12-h light/dark cycle and provided with food and water ad libitum. No more than five mice were housed in each cage.

### Establishment of mouse obesity (positive energy balance) model

Mice were randomly assigned to a normal diet (ND, *n* = 16), and the remaining mice were fed an HFD (*n* = 54). The nutritional formulas of ND and HFD were previously reported [22], and the feed was provided by Jianmin Company Ltd. (Shenyang, China). After 10 weeks of feeding, five obesity-resistant mice were removed from the HFD group, whereas the others reached a weight of more than 120% that of ND mice, meeting the animal obesity model standard [23]. Five obesity-resistant mice were removed from the HFD group, and the obese mice were randomly assigned to three groups: obesity control (OC, *n* = 16), obesity moderate-volume exercise (OME, *n* = 16), and obesity heavy-volume exercise (OHE, *n* = 17). No significant differences in body weight were observed among the three groups (*P* > 0.05). The ND group was used as the normal control (NC). Mice in the OME and OHE groups were subjected to swimming intervention for 8 weeks.

### Exercise intervention (negative energy balance)

Mice in the OME and OHE groups were allowed to swim freely in a plastic pool with a diameter of 45 cm, water depth of 60 cm, and water temperature of 32 ± 1 °C. The exercise program was previously reported [22]. The formal 8-week swimming training session began after 2 days of an adaptive swimming session. Training was performed with increasing exercise loads. The initial load of the OME group was 20 min × 1 time/day and that of the OHE group was 20 min × 2 times/day, and the interval between the two exercises in the OHE group was 6 h. In the first and second weeks, the exercise period was increased by 10 min every day. By the end of the second week, the exercise times of the OME and OHE groups were 120 min once per day and 120 min twice per day, respectively. This load was maintained for 6 weeks.

### Sample collection

To evaluate the adaptive response to long-term exercise, the selection time was 36–40 h after the last exercise in the OME and OHE groups. All groups were selected after fasting for 12 h to exclude the effects of the diet on the measured indicators. Anesthesia was induced by intraperitoneal injection of sodium pentobarbital (50 mg/kg body weight, Sinopharm Group Chemical Reagent Co., Ltd.). Blood was collected from the orbital venous plexus, centrifuged for 20 min (4 °C, 900×*g*) to separate the serum, and stored at − 80 °C. The serum hormone index was measured. The isolated hypothalamus was frozen in liquid nitrogen and then transferred to − 80 °C until subsequent experiments. The fat around the testis and kidneys was separated, and the intra-abdominal mesenteric adipose depot was dissected. An electronic balance was used to weigh the fat content in the abdominal cavity of the mice. The hypothalamus samples from each group were divided into two parts: one for real-time polymerase chain reaction (PCR) (*n* = 8) and the other for western blotting (*n* = 8). The number of samples of the other indicators was also *n* = 8 for both PCR and western blotting.

### Measurement of gonad morphological indicators

The testis, epididymis, and seminal vesicles of the mice were quickly separated and weighed. Testis coefficient = bilateral testis weight (g)/body weight (g); seminal vesicle coefficient = bilateral seminal vesicle gland weight (g)/body weight (g); epididymis coefficient = bilateral epididymal weight (g)/body weight (g).

### Cauda epididymal sperm count and measurement of sperm motility and apoptosis

After weighing, the epididymis was quickly placed in 1.0 mL of saline solution. The epididymis tail was cut into three segments with scissors, pressed gently to release the semen from the vas deferens, and filtered through a layer of mirror paper to prepare the sperm suspension. This suspension was then incubated in saline at 37 °C for 10 min. Approximately 10 μL of the diluted sperm suspension was transferred to each counting chamber of the hemocytometer and allowed to stand for 5 min. The count of motile and immotile sperms in these aliquots was determined. Motility was defined as a sperm showing any movement (fast linear motion, slow or slow linear or nonlinear motion, in situ motion) in the flagellum during a 30-s observation period. Sperm motility was expressed as a percentage of motile sperm to total sperm [24].

The remaining portion of the sperm suspension was used to measure sperm apoptosis. The sperm suspension was centrifuged at 400×*g* for 5 min to remove the supernatant and collect the sperm. Approximately 2 mL phosphate buffer was added to clean the sperm. The sample was then centrifuged (400×*g* for 5 min), and the supernatant was removed. The sperm suspension was prepared at a concentration of 0.25–1.0 × 10^7^ sperm/mL with annexin V binding buffer. Next, 100 μL sperm suspension was transferred to a 1.5-mL test tube, to which 5 μL FITC annexin V and 10 μL propidium iodide solution were added. The sperm were gently vortexed and incubated at room temperature (25 °C) in the dark for 15 min. After adding 400 μL annexin V binding buffer to each tube, the sperm was detected with a flow cytometer (Cytoflex; Beckman Coulter, Brea, CA, USA). The data were processed using Cytexpert software, and the percentage of early apoptotic sperm within the total sperm of each group was calculated.

### Determination of serum hormone

The FSH, LH, TNF-α, IL-10, testosterone (T), and estradiol (E2) in the serum of mice were determined by enzyme-linked immunosorbent assay. The kits were purchased from Shanghai Zylian Biotechnology Co., Ltd. (Shanghai, China). A double-antibody sandwich method was used to determine the levels of the corresponding hormones (FSH, LH, T, E2, TNF-α, IL-10) in the mouse samples according to the manufacturer’s instructions. The absorbance (optical density value) was measured using a microplate reader at 450 nm, and the leptin, FSH, LH, T, E2, TNF-α, and IL-10 levels in the mouse samples were calculated from a standard curve.

### Isolation of RNA and real-time PCR analysis

Total RNA from the mouse hypothalamus was extracted using RNA extraction reagent (Nuoweizan Biotechnology Co., Ltd., Nanjing, China) according to the kit instructions. Next, 1 μg of total RNA was reverse-transcribed into cDNA using a reverse transcription kit (Promega, Madison, WI, USA) using a 96-well thermal cycler (Applied Biosystems, Foster City, CA, USA). Finally, a real-time amplification PCR kit (Promega) was used to determine the content of target mRNA with a real-time amplification PCR instrument (Applied Biosystems) according to the instructions. The sequences of the target gene primers are shown in Table 1 (supplementary file). All primers were designed and synthesized by Shanghai Biotech Engineering Co., Ltd. (Shanghai, China). For each sample, three reactions were performed, and Actb was used as an internal reference. The expression values were calculated by the 2^-△△Ct^ method.

**Table 1:**
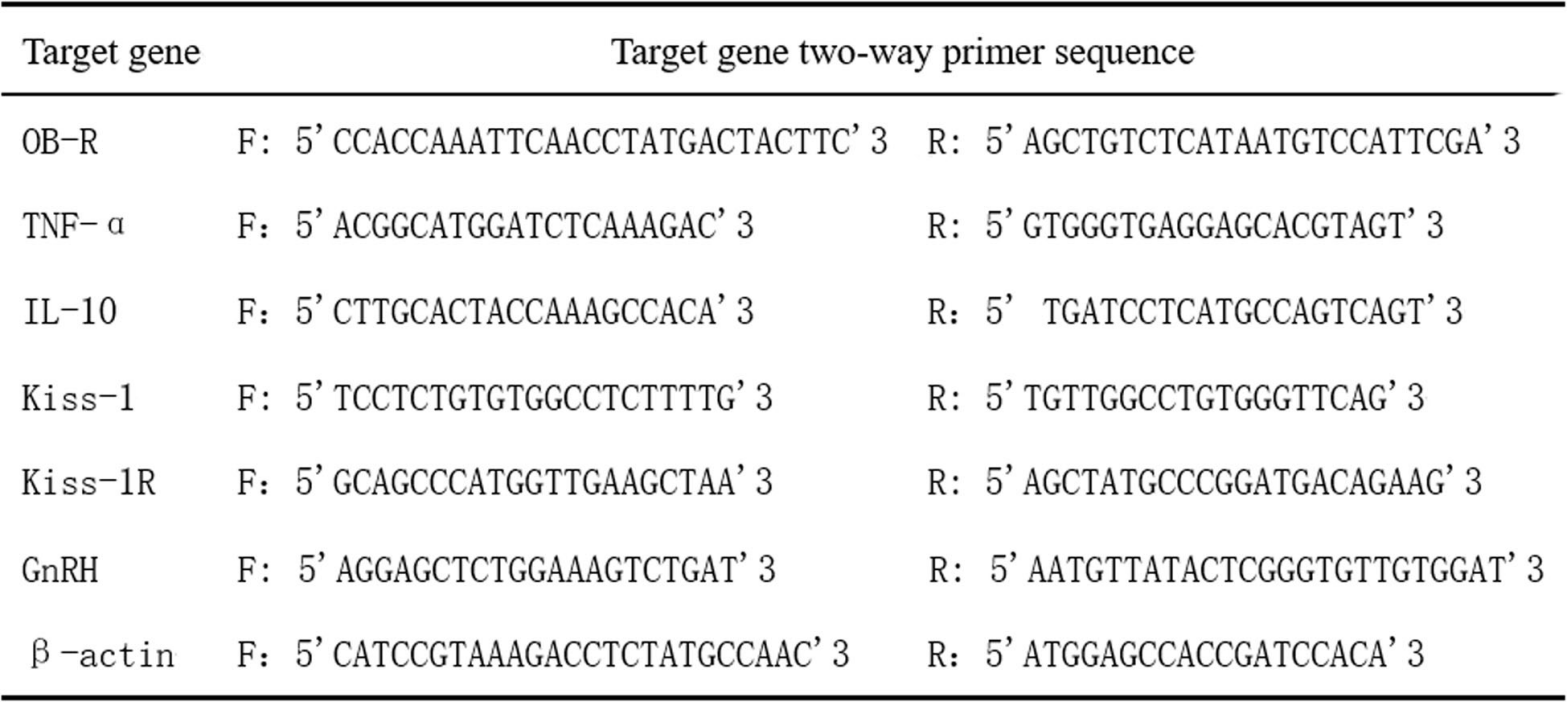
R-T PCR reactiion primer sequences

### Western blotting

The mouse hypothalamus was weighed and homogenized in radioimmunoprecipitation assay buffer supplemented with a protease inhibitor (phenylmethylsulfonyl fluoride). The homogenate was placed in an ice bath and centrifuged. The supernatant was collected, and a bicinchoninic acid protein assay kit was used for protein quantification (Ding Guo Changsheng Biotechnology Co., Ltd., Beijing, China) using a microplate reader (Thermo Fisher Scientific, Waltham, MA, USA). The target protein was separated by sodium dodecyl sulfate polyacrylamide gel electrophoresis, by loading 30–50 μg of protein lysate in each well. The proteins were transferred to a nitrocellulose membrane, which was blocked with 5% skim milk powder blocking solution for 1 h, and incubated with a suitable concentration of the following primary antibodies at 4 °C overnight (12 h): anti-LepR (Santa Cruz Biotechnology, Dallas, TX, USA, sc-8391), TNF-α (Cell Signaling Technology, Danvers, MA, USA, 11948), IL-10 (ABclonal, Wuhan, China, A2171), kisspeptin (Santa Cruz Biotechnology, sc-101,246), anti-GPR54 (Abcam, Cambridge, UK, ab100896), anti-GnRH (Abcam, ab16216), and anti-β-actin (ABclonal, AC026). The membrane was then incubated for 1 h at room temperature with a fluorescent secondary antibody (IRDye 800CW goat anti-rabbit, LI-COR, USA; HRP Goat Anti-Mouse IgG (H + L), ABclonal, AS003) diluted to 1:15,000. Finally, the nitrocellulose membrane strip was placed in an Odyssey infrared fluorescence scanning imaging system (Li-Cor, Lincoln, NE, USA), and Image Studio software was used to quantitatively analyze the protein bands. Finally, the target protein/β-actin ratio was calculated.

### Statistical analysis

The data are expressed as the mean ± standard error. Multiple-group comparisons were performed by one-way analysis of variance followed by Student–Newman–Keuls post-hoc test for multiple comparisons. The results were considered significant for *P*-values < 0.05. The analyses were performed using SPSS 18.0 software (SPSS, Inc., Chicago, IL, USA).

## Results

### Influence of HFD and exercise on body weight and abdominal fat content

**Fig. 1:**
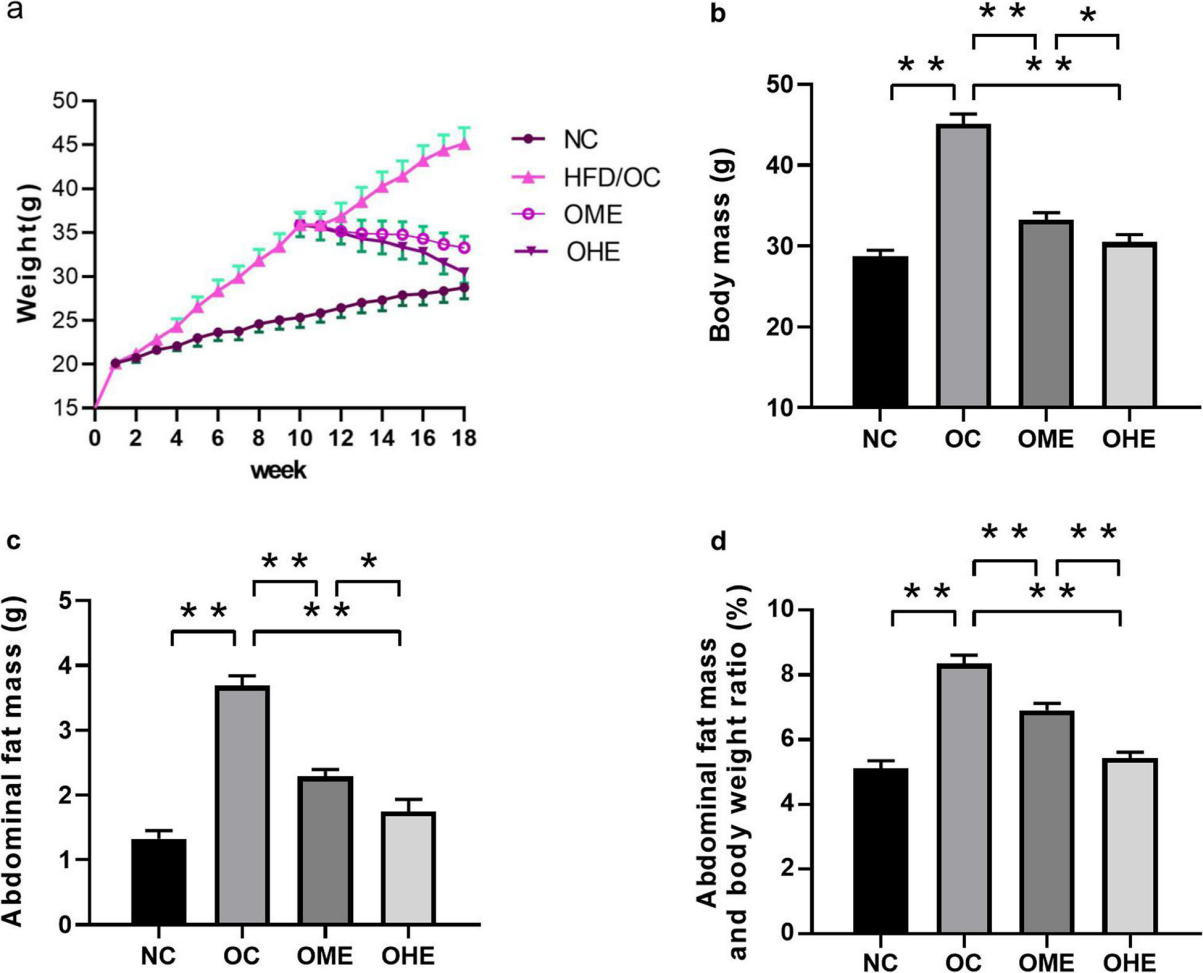
Influence of high-fat diet and exercise on body weight and abdominal fat content. Values are reported as the mean ± standard error (SE). NC, normal control; OC, obesity control; OME, obesity moderate-load exercise; OHE, obesity high-load exercise; vs. *P < 0.05, **P < 0.01

The body weight changes observed in the HFD groups at 18 weeks are shown in Fig. 1a. After 18 weeks of HFD feeding, the body weight (Fig. 1b), abdominal fat content (Fig. 1c), and lipid ratio of OC mice (Fig. 1d) were significantly higher than those of NC mice. After 8 weeks of exercise intervention, the body weight (Fig. 1b), abdominal fat content (Fig. 1c), and lipid ratio of mice (Fig. 1d) in the OME and OHE groups were significantly lower than those of mice in the OC group; these changes were more pronounced in the OHE group than in the OME group (Fig. 1b–d).

### Effects of HFD and exercise on the weight of reproductive organs in male mice

**Fig. 2:**
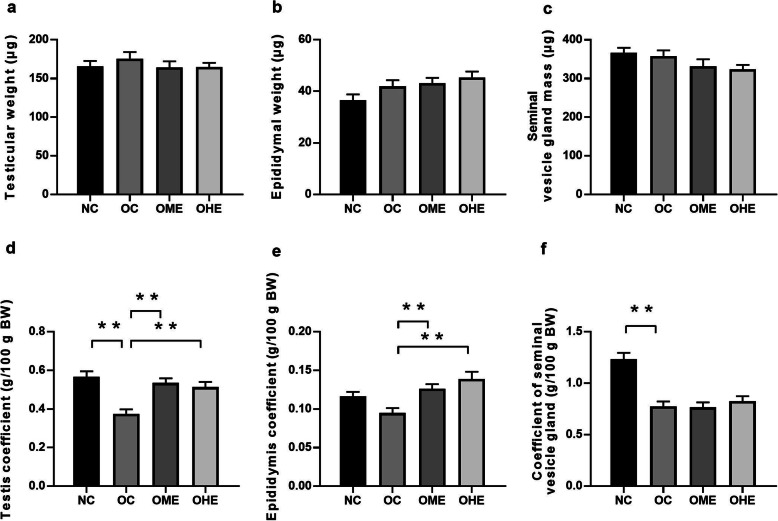
Effects of high-fat diet and exercise on the weight of reproductive organs in male mice. Values are reported as the mean ± standard error (SE). NC, normal control; OC, obesity control; OME, obesity moderate-load exercise; OHE, obesity high-load exercise; vs. *P < 0.05, **P < 0.01

There were no significant differences in the weight of the testis (Fig. 2a), epididymis (Fig. 2b), and seminal vesicles (Fig. 2c) among mice in the NC, OC, OME, and OHE groups. However, the testis coefficient (Fig. 2d) and seminal vesicle coefficient (Fig. 2f) of the OC group were significantly lower than those of the NC group. After 8 weeks of moderate- and high-volume exercise, the testis coefficient (Fig. 2d) and epididymis coefficient (Fig. 2) of the OME and OHE groups were significantly higher than those of the OC group and were restored to normal levels.

### Effects of HFD and exercise on serum leptin and sex hormones in male mice

**Fig. 3:**
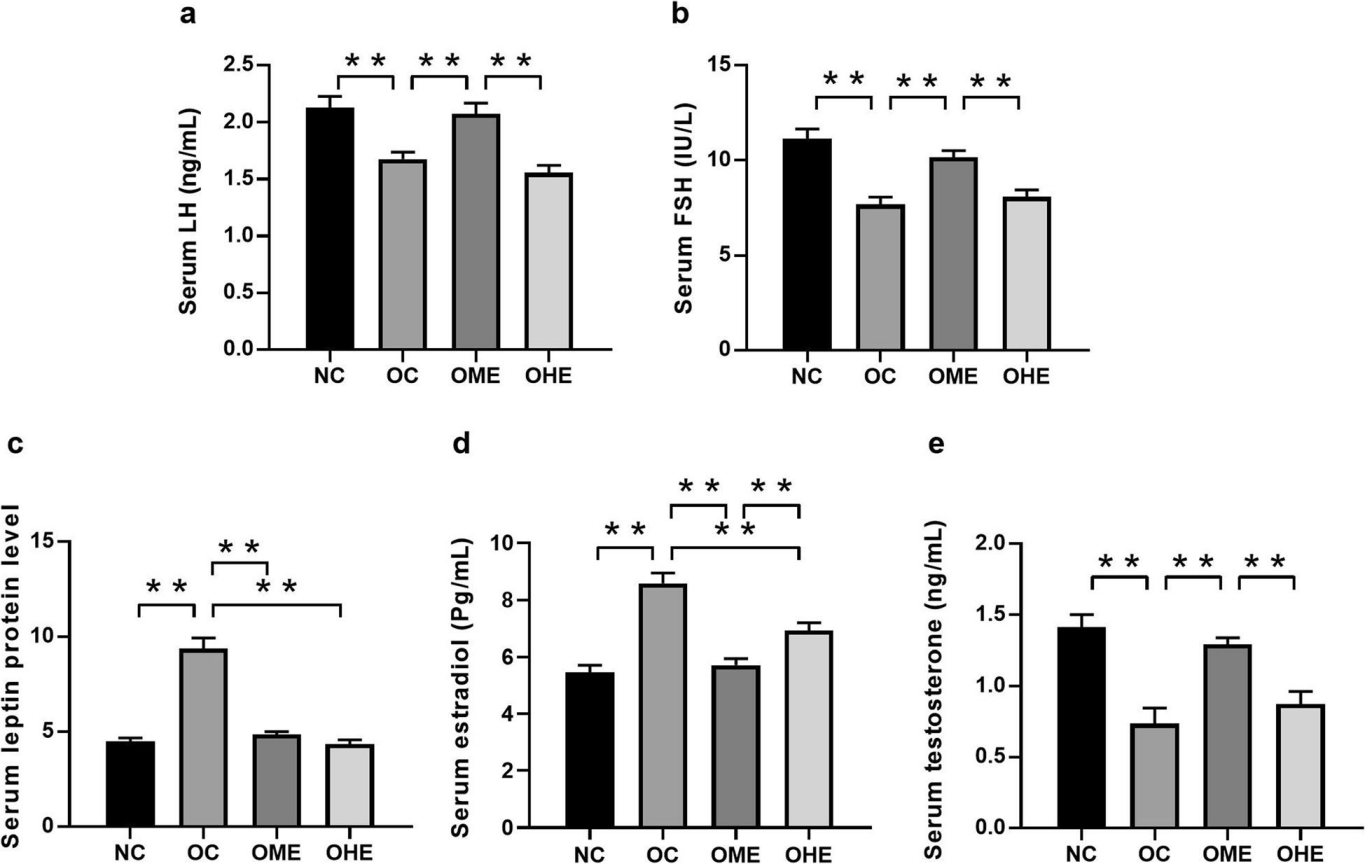
Effects of high-fat diet and exercise on serum leptin and sex hormones in male mice. Values are reported as the mean ± standard error (SE). NC, normal control; OC, obesity control; OME, obesity moderate-load exercise; OHE, obesity high-load exercise; vs. *P < 0.05, **P < 0.01

The serum levels of leptin (Fig. 3c) and E2 (Fig. 3d) were significantly increased, whereas those of LH (Fig. 3a), FSH (Fig. 3b), and T (Fig. 3e) levels were significantly decreased in the OC group compared with those in the NC group (Fig. 3). After 8 weeks of exercise intervention, leptin (Fig. 3c) and E2 (Fig. 3d) levels were significantly decreased, whereas LH (Fig. 3a), FSH (Fig. 3b), and T (Fig. 3e) levels were significantly increased in the OME group compared with those in the OC group (Fig. 3). The examined indicators did not change significantly in the OHE group, but the LH (Fig. 3a), FSH (Fig. 3b), and T (Fig. 3e) levels significantly differed between the OME and OHE groups.

### Effects of HFD and exercise on serum TNF-α and IL-10 in male mice

**Fig. 4:**
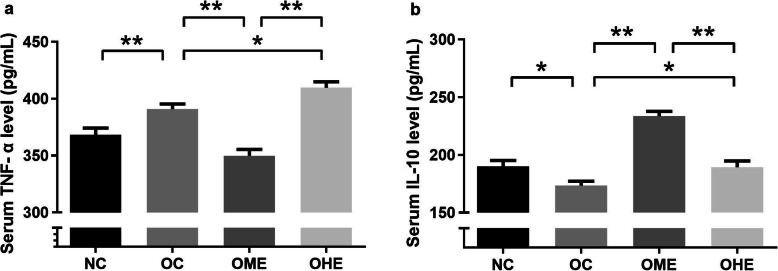
Effects of high-fat diet and exercise on serum TNF-α and IL-10 in male mice. Values are reported as the mean ± standard error (SE). NC, normal control; OC, obesity control; OME, obesity moderate exercise; OHE, obesity heavy loads exercise; vs. *P < 0.05, **P < 0.01

The serum level of TNF-α was significantly increased, whereas that of IL-10 was significantly decreased in the OC group compared with those in the NC group (Fig. 4). After 8 weeks of exercise intervention (Fig. 4a), the TNF-α level significantly decreased, whereas the IL-10 level significantly increased in the OME group compared with those in the OC group (Fig. 4b). In contrast, the TNF-α level was significantly increased and IL-10 level was significantly decreased in the OHE group compared with those in the OC group (Fig. 4a, b).

### Effect of HFD and exercise on sperm quality in male mice

**Fig. 5:**
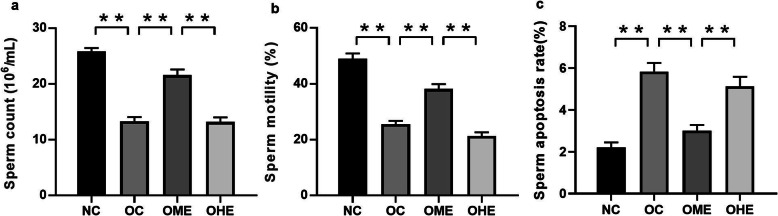
Effect of high-fat diet and exercise on sperm quality in male mice. Values are reported as the mean ± standard error (SE). NC, normal control; OC, obesity control; OME, obesity moderate-load exercise; OHE, obesity high-load exercise; vs. *P < 0.05, **P < 0.01

**Fig. 6:**
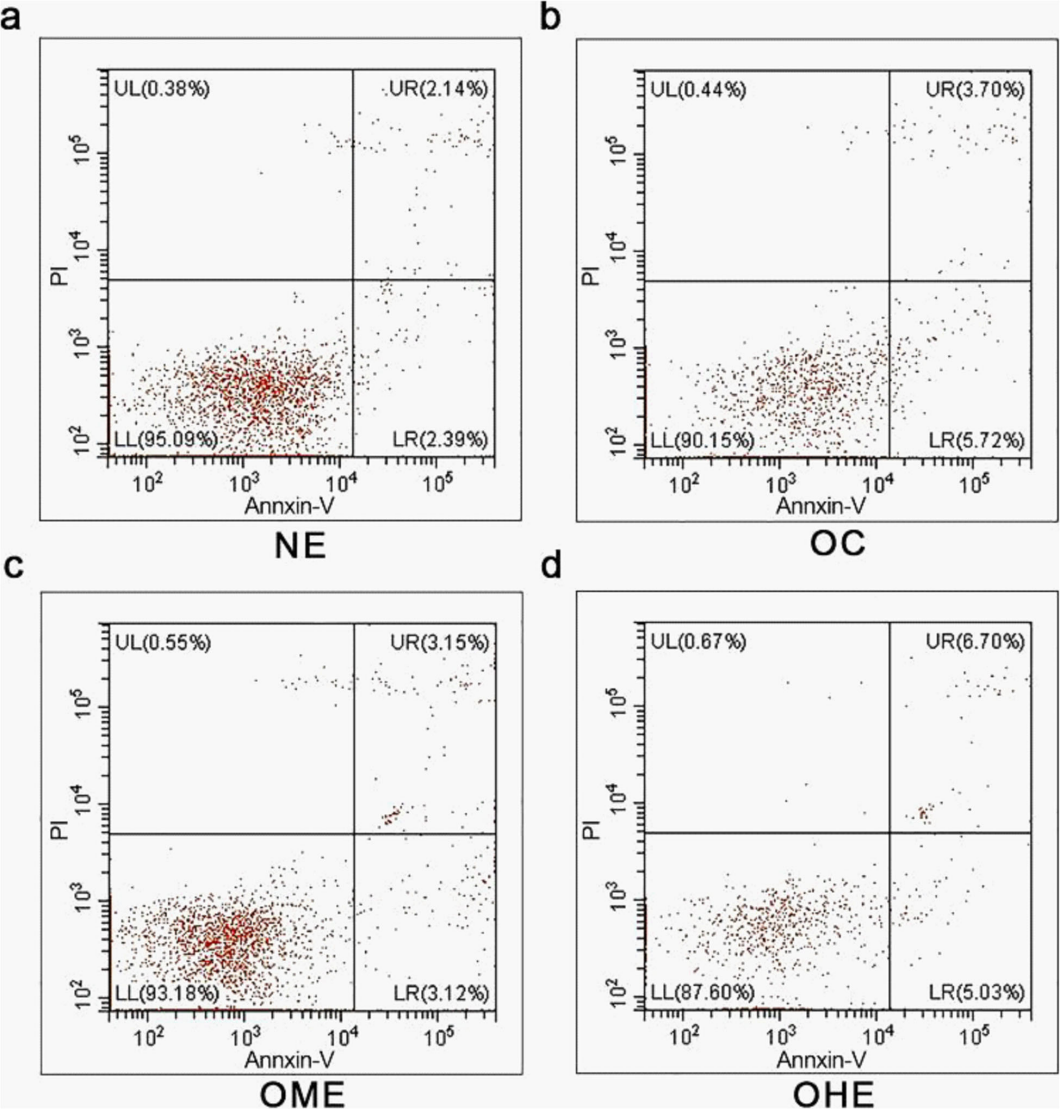
Effect of high-fat diet and exercise on sperm apoptosis rate in male mice. **a** Normal control (NC) group; **b** high-fat diet obesity control (OC) group; **c** obesity moderate-load exercise (OME) group; **d** obesity high-load exercise (OHE) group

The sperm count (Fig. 5a) and sperm motility (Fig. 5b) significantly decreased, and the sperm apoptosis rate (Fig. 5c and 6b) significantly increased in the OC group compared to those in the NC group. After 8 weeks of exercise intervention, the count and motility of sperm in the OME group increased significantly (Fig. 5a–b), and the sperm apoptosis rate (Fig. 5c and 6b–d) decreased significantly compared with those in the OC group. Mice in the OHE group performed two exercise sessions per day with 6 h between each one; the sperm count (Fig. 5a) and sperm motility (Fig. 5b) were reduced, and apoptosis was increased (Fig. 5c). However, there was no significant difference between the OHE group and OC group (Fig. 5a–c and 6a–d).

### Effects of HFD and exercise on expression of the leptin receptor, TNF-α, IL-10, kiss1, kiss1R, and GnRH in the hypothalamus of male mice

**Fig. 7:**
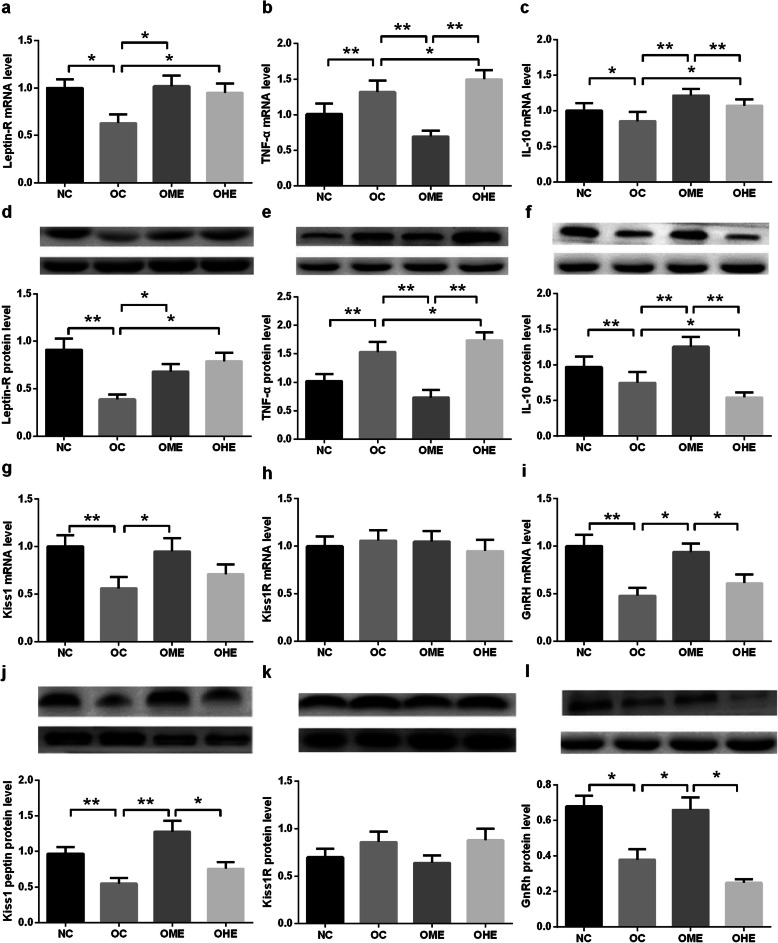
Effects of high-fat diet and exercise on various molecules in the hypothalamus of male mice. The expression of leptin receptor, TNF-α, IL-10, kiss1, kiss1R, and GnRH in the hypothalamus of male mice was determined. The values are reported as the mean ± standard error (SE). NC, normal control; OC, obesity control; OME, obesity moderate-load exercise; OHE, obesity high-load exercise, vs. *P < 0.05, **P < 0.01

The mRNA and protein expression levels of the leptin receptor, IL-10, kiss, and GnRH in the hypothalamus were significantly lower and those of TNF-α were significantly higher (Fig. 7) in the OC group than in the NC group. Notably, after 8 weeks of exercise intervention, a significant decrease was observed in the expression of TNF-α, whereas the expression levels of the leptin receptor, IL-10, kiss, and GnRH increased significantly in the OME group. In contrast, in the OHE group, hypothalamic leptin receptor and TNF-α were significantly upregulated (Fig. 7), and IL-10 was significantly decreased, with the other indicators showing no significant change (Fig. 7). A significant difference was observed in the protein expression of kiss (Fig. 7) and mRNA and protein expression of TNF-α, IL-10, and GnRH (Fig. 7) between the OME and OHE groups. There was no significant difference in *kiss1R* mRNA and protein expression among groups.

## Discussion

The effect of a positive energy balance on male reproduction is related to the degree of obesity. Being overweight had no effect on androgen and sperm quality [25]; however, when the body mass index of obese men was more than 30 kg/m^2^, the risk of hypogonadal dystrophy increased by 8-fold [26]. In this study, the male mice began to eat an HFD (positive energy balance) at an early stage of development. After 18 weeks, the weight of mice in the obese group was 1.4-fold higher than that of the mice in the normal group. The coefficient of reproductive organs, level of serum testosterone, and quality of sperm decreased, and the rate of sperm apoptosis and estradiol levels increased in obese mice, suggesting that a positive energy balance at an early stage of development inhibits the development of reproductive organs, resulting in hypogonadism in males.

The secretion of testosterone and spermatogenesis are mainly regulated by the HPG axis. Decreased serum testosterone levels in obese mice are related to LH [27]. The expression of *GnRH* mRNA and number of immunopositive cells in the hypothalamus of obese male rabbits were reported to decrease [28]. Our results also confirmed that the expression of *GnRH* mRNA and protein in the hypothalamus of obese mice decreased significantly, accompanied by downregulation of serum LH, FSH, and T. It further suggests that obesity may cause HH in male mice. However, the mechanism is unclear. In this study, we found that serum leptin and TNF-α levels increased, whereas the levels of anti-inflammatory factor IL-10 decreased in obese mice. Our previous studies confirmed that the serum leptin level is negatively correlated with the testosterone level in obese male mice [22]. The decrease in testosterone levels is related to increased proinflammatory cytokines [29]. Therefore, the HH symptoms in obese rats may be related to leptin resistance and the chronic inflammatory response.

Leptin can regulate the energy balance and reproduction. However, GnRH neurons do not/rarely express the leptin receptor [30, 31]. Therefore, leptin cannot regulate GnRH neurons directly [32]. Some studies have shown that mouse arcuate nucleus neurons co-express OB-R and kisspeptin. Further, mutations in kisspeptin or its receptor can stop mice and humans from reaching puberty and lead to the development of HH and infertility [33]. Treating *ob*/*ob* mice with leptin increased the expression of *KiSS1* in the hypothalamus and induced sexual maturation. The decrease of OB-R and kisspeptin expression in the mouse hypothalamus is related to sexual development and reproductive dysfunction of males [34]. However, studies have also shown that obesity induced by HFD does not affect the expression of *kiss-gpr54* mRNA in the hypothalamus [35]. The reason for the inconsistent results of leptin on HPG may be related to the degree of the positive energy balance in obesity [36, 37]. Our study confirmed that HFD-induced positive energy obese mice showed increased levels of plasma leptin and downregulated mRNA and protein expression of the leptin receptor, kiss, and GnRH in the hypothalamus. Based on these results, a decrease in leptin receptors in the hypothalamus may be alleviated by high serum leptin, which may limit the transmission of the leptin signal to kiss neurons, inhibit the activation of GnRH by kisspeptin, and lead to a decrease in HPG axis function.

Recent studies have shown that LPS can reduce the expression of *KiSS1* mRNA in the hypothalamus and inhibit LH [38, 39] in female rats. In vitro experiments also showed that TNF-α can reduce GnRH secretion by downregulating kisspeptin signal transduction [19]. In this study, we found that TNF-α expression in the hypothalamus increased significantly, whereas the expression of IL-10 decreased. The decrease in the expression of kiss and GnRH in the hypothalamus and decrease of gonadal function suggested that a long-term positive energy balance inhibits the stimulation of kiss neurons on the HPG axis through leptin resistance and the inflammatory response. If the long-term positive energy balance leads to improvement in leptin resistance and the inflammatory response, it is unclear whether kisspeptin can alleviate the inhibition of GnRH, and thus further studies are needed.

To further verify the effect of leptin and the inflammatory response on kiss and GnRH, the positive energy balance that was the result of the intake of an HFD was reversed by 8 weeks of moderate-load exercise. It was found that the body weight, body fat, serum leptin, and TNF-α were significantly reduced following moderate-load exercise. Meanwhile, the levels of serum IL-10, FSH, LH, and testosterone were significantly increased, the sperm apoptosis rate was decreased, and the Sperm count and motility were increased, indicating that body fat was effectively reduced. This suggested that moderate-load exercise improved leptin resistance and inflammatory reaction and alleviated the HH symptoms caused by obesity. Although heavy-load exercise also significantly reduced body weight, body fat, and serum leptin, it increased TNF-α and decreased IL-10 in serum and the hypothalamus. There were no significant changes in the related Indicators of male reproductive function, FSH, LH, and sperm quality parameters, suggesting that although heavy-load exercise also improved physical fitness and leptin resistance, it increased the inflammatory response and had no significant effect on alleviating the HH symptoms caused by obesity. Previous studies have shown that exercise load is closely related to the leptin level, inflammatory response, and male reproduction [22, 29]. Moderate-load exercise can effectively reduce the inflammatory response caused by obesity, improve leptin resistance [22], increase serum testosterone levels in men and rats/mice, and improve the quality of sperm [39]. However, heavy-load or high-intensity exercise increased the inflammatory response [21] and decreased the levels of serum LH, FSH, testosterone, and sperm quality [40, 41]. This suggests that the different effects of different loads of exercise on the reproductive function of obese males may be related to the improvement in leptin resistance and the inflammatory response. It remains unclear whether the mechanism of action is related to the regulation of the hypothalamic kiss on the HPG axis.

Few studies have examined the effect of exercise on the HPG axis of the hypothalamic kiss, and the results are controversial. Recent studies showed that the expression of GnRH in the hypothalamus of normal rats subjected to moderate- and high-intensity exercise remains unchanged and decreased, respectively, but there is no effect on the expression of kiss [42]. However, proper exercise can effectively reverse the inhibition of HFD-induced metabolic syndrome on hypothalamic kiss and GnRH in rabbits [43]. Our results showed that after 8 weeks of moderate-load exercise, the mRNA and protein expression of the leptin receptor, IL-10, kiss, and GnRH in the hypothalamus was upregulated and the expression of TNF-α was downregulated, which were consistent with the improvement in HH symptoms. Thus, moderate-load exercise may improve leptin resistance and inflammation in the hypothalamus, upregulate kiss expression, and reverse the inhibition of positive energy balance in male HPG and HH symptoms. Although high-load exercise decreased the serum leptin level and increased expression of the leptin receptor in the hypothalamus, TNF-α in the hypothalamus increased significantly, IL-10 decreased significantly, and no changes were observed in kiss and GnRH, which were consistent with the trends in serum sex hormone and sperm quality changes. Previous studies have showed that overtraining can cause changes in TNF-α and IL-10 in the hypothalamus, increases the levels of inflammatory factors, and decreases the levels of anti-inflammatory factors [21]. Our results suggest that high-load exercise aggravates the inflammatory response of the hypothalamus caused by obesity and counteracts the upregulation of kisspeptin in the hypothalamus by improving leptin resistance. Therefore, when using exercise intervention to improve obese HH in the clinic, the exercise load should be controlled to avoid inflammatory reactions caused by an excessive load and offset or reduce the beneficial effect of leptin resistance improvement on reproductive function in obese men.

## Conclusion

The in vivo positive energy balance induced leptin resistance and inflammation in mice, downregulated kisspeptin expression in the hypothalamus, inhibited the function of the HPG axis, and induced HH. Moderate-load exercise reversed the positive energy balance, improved leptin resistance and inflammation, upregulated kiss expression, and alleviated HPG function and HH symptoms. Heavy-load exercise increased inflammation, counteracted leptin resistance, and did not effectively relieve the obesity-induced HH symptoms. The exact mechanism of these effects must be verified in vitro.

## Data Availability

Not applicable.

## Abbreviations

FSH: Follicle-stimulating hormone
HFD: High-fat diet
HH: Hypogonadotropic hypogonadism
HPG: Hypothalamic-pituitary-gonadal
HPT: Hypothalamic-pituitary-testis
LH: Luteinizing hormone
TNF-α: Tumor necrosis factor-alpha
NC: Normal control
ND: Normal diet
OC: Obesity control
OHE: Obesity heavy-volume exercise
OME: Obesity moderate-volume exercise
SE: Standard error

## References

[1] Frisch RE, McArthur JW (1974). Menstrual cycles: fatness as a determinant of minimum weight for height necessary for their maintenance or onset. Science..

[2] Compagnucci C, Compagnucci GE, Lomniczi A, Mohn C, Vacas I, Cebral E (2002). Effect of nutritional stress on the hypothalamo-pituitary-gonadal axis in the growing male rat. Neuroimmunomodulation..

[3] Corona G, Vignozzi L, Sforza A, Mannucci E, Maggi M (2015). Obesity and late-onset hypogonadism. Mol Cell Endocrinol.

[4] Pivonello R, Menafra D, Riccio E, Garifalos F, Mazzella M, de Angelis C (2019). Metabolic disorders and male hypogonadotropic hypogonadism. Front Endocrinol.

[5] Hajizadeh Maleki B, Tartibian B, Eghbali M, Asri-Rezaei S (2013). Comparison of seminal oxidants and antioxidants in subjects with different levels of physical fitness. Andrology..

[6] Laing BT, Do K, Matsubara T, Wert DW, Avery MJ, Langdon EM (2016). Voluntary exercise improves hypothalamic and metabolic function in obese mice. J Endocrinol.

[7] Maresh CM, Whittlesey MJ, Armstrong LE, Yamamoto LM, Judelson DA, Fish KE (2006). Effect of hydration state on testosterone and cortisol responses to training-intensity exercise in collegiate runners. Int J Sports Med.

[8] Jana K, Dutta A, Chakraborty P, Manna I, Firdaus SB, Bandyopadhyay D (2014). Alpha-lipoic acid and N-acetylcysteine protects intensive swimming exercise-mediated germ-cell depletion, pro-oxidant generation, and alteration of steroidogenesis in rat testis. Mol Reprod Dev.

[9] Yi X, Tang D, Cao S, Li T, Gao H, Ma T (2020). Effect of different exercise loads on testicular oxidative stress and reproductive function in obese male mice. Oxidative Med Cell Longev.

[10] De Bond JA, Smith JT (2014). Kisspeptin and energy balance in reproduction. Reproduction..

[11] Irwig MS, Fraley GS, Smith JT, Acohido BV, Popa SM, Cunningham MJ (2004). Kisspeptin activation of gonadotropin releasing hormone neurons and regulation of KiSS-1 mRNA in the male rat. Neuroendocrinology..

[12] Han SK, Gottsch ML, Lee KJ, Popa SM, Smith JT, Jakawich SK (2005). Activation of gonadotropin-releasing hormone neurons by kisspeptin as a neuroendocrine switch for the onset of puberty. J Neurosci.

[13] Smith JT, Li Q, Pereira A, Clarke IJ (2009). Kisspeptin neurons in the ovine arcuate nucleus and preoptic area are involved in the preovulatory luteinizing hormone surge. Endocrinology..

[14] Gottsch ML, Cunningham MJ, Smith JT, Popa SM, Acohido BV, Crowley WF (2004). A role for kisspeptins in the regulation of gonadotropin secretion in the mouse. Endocrinology..

[15] Castellano JM, Navarro VM, Fernandez-Fernandez R, Nogueiras R, Tovar S, Roa J (2005). Changes in hypothalamic KiSS-1 system and restoration of pubertal activation of the reproductive axis by kisspeptin in undernutrition. Endocrinology..

[16] Venancio JC, Margatho LO, Rorato R, Rosales RRC, Debarba LK, Coletti R (2017). Short-term high-fat diet increases leptin activation of CART neurons and advances puberty in female mice. Endocrinology..

[17] Lee CY, Li S, Li XF, Stalker DAE, Cooke C, Shao B (2019). Lipopolysaccharide reduces gonadotrophin-releasing hormone (GnRH) gene expression: role of RFamide-related peptide-3 and kisspeptin. Reprod Fertil Dev.

[18] Iwasa T, Matsuzaki T, Murakami M, Shimizu F, Kuwahara A, Yasui T (2008). Decreased expression of kisspeptin mediates acute immune/inflammatory stress-induced suppression of gonadotropin secretion in female rat. J Endocrinol Investig.

[19] Sarchielli E, Comeglio P, Squecco R, Ballerini L, Mello T, Guarnieri G (2017). Tumor necrosis factor-alpha impairs kisspeptin signaling in human gonadotropin-releasing hormone primary neurons. J Clin Endocrinol Metab.

[20] Watanobe H, Hayakawa Y (2003). Hypothalamic interleukin-1 beta and tumor necrosis factor-alpha, but not interleukin-6, mediate the endotoxin-induced suppression of the reproductive axis in rats. Endocrinology..

[21] Pereira BC, da Rocha AL, Pauli JR, Ropelle ER, de Souza CT, Cintra DE (2015). Excessive eccentric exercise leads to transitory hypothalamic inflammation, which may contribute to the low body weight gain and food intake in overtrained mice. Neuroscience..

[22] Yi X, Gao H, Chen D, Tang D, Huang W, Li T (2017). Effects of obesity and exercise on testicular leptin signal transduction and testosterone biosynthesis in male mice. Am J Physiol Regul Integr Comp Physiol.

[23] Giovambattista A, Suescun MO, Nessralla CC, Franca LR, Spinedi E, Calandra RS (2003). Modulatory effects of leptin on leydig cell function of normal and hyperleptinemic rats. Neuroendocrinology..

[24] Yokoi K, Uthus EO, Nielsen FH (2003). Nickel deficiency diminishes sperm quantity and movement in rats. Biol Trace Elem Res.

[25] Duale N, Steffensen IL, Andersen J, Brevik A, Brunborg G, Lindeman B (2014). Impaired sperm chromatin integrity in obese mice. Andrology..

[26] Tajar A, Forti G, O'Neill TW, Lee DM, Silman AJ, Finn JD (2010). Characteristics of secondary, primary, and compensated hypogonadism in aging men: evidence from the European male ageing study. J Clin Endocrinol Metab.

[27] Xu X, Wang L, Luo D, Zhang M, Chen S, Wang Y (2018). Effect of testosterone synthesis and conversion on serum testosterone levels in obese men. Horm Metab Res.

[28] Corona G, Rastrelli G, Monami M, Saad F, Luconi M, Lucchese M (2013). Body weight loss reverts obesity-associated hypogonadotropic hypogonadism: a systematic review and meta-analysis. Eur J Endocrinol.

[29] Chen D, Cao S, Chang B, Ma T, Gao H, Tong Y (2019). Increasing hypothalamic nucleobindin 2 levels and decreasing hypothalamic inflammation in obese male mice via diet and exercise alleviate obesity-associated hypogonadism. Neuropeptides..

[30] Finn PD, Cunningham MJ, Pau KY, Spies HG, Clifton DK, Steiner RA (1998). The stimulatory effect of leptin on the neuroendocrine reproductive axis of the monkey. Endocrinology..

[31] Watanobe H (2002). Leptin directly acts within the hypothalamus to stimulate gonadotropin-releasing hormone secretion in vivo in rats. J Physiol.

[32] Park HK, Ahima RS (2015). Physiology of leptin: energy homeostasis, neuroendocrine function and metabolism. Metabolism..

[33] Topaloglu AK, Tello JA, Kotan LD, Ozbek MN, Yilmaz MB, Erdogan S (2012). Inactivating KISS1 mutation and hypogonadotropic hypogonadism. N Engl J Med.

[34] Zhai L, Zhao J, Zhu Y, Liu Q, Niu W, Liu C (2018). Downregulation of leptin receptor and kisspeptin/GPR54 in the murine hypothalamus contributes to male hypogonadism caused by high-fat diet-induced obesity. Endocrine..

[35] Dudek M, Kolodziejski PA, Pruszynska-Oszmalek E, Sassek M, Ziarniak K, Nowak KW (2016). Effects of high-fat diet-induced obesity and diabetes on Kiss1 and GPR54 expression in the hypothalamic-pituitary-gonadal (HPG) axis and peripheral organs (fat, pancreas and liver) in male rats. Neuropeptides..

[36] Hofny ER, Ali ME, Abdel-Hafez HZ, Kamal Eel D, Mohamed EE, Abd El-Azeem HG (2010). Semen parameters and hormonal profile in obese fertile and infertile males. Fertil Steril.

[37] Zhao J, Zhai L, Liu Z, Wu S, Xu L (2014). Leptin level and oxidative stress contribute to obesity-induced low testosterone in murine testicular tissue. Oxidative Med Cell Longev.

[38] Iwasa T, Matsuzaki T, Tungalagsuvd A, Munkhzaya M, Kawami T, Niki H (2014). Hypothalamic Kiss1 and RFRP gene expressions are changed by a high dose of lipopolysaccharide in female rats. Horm Behav.

[39] Rosety MA, Diaz AJ, Rosety JM, Pery MT, Brenes-Martin F, Bernardi M (2017). Exercise improved semen quality and reproductive hormone levels in sedentary obese adults. Nutr Hosp.

[40] Safarinejad MR, Azma K, Kolahi AA (2009). The effects of intensive, long-term treadmill running on reproductive hormones, hypothalamus-pituitary-testis axis, and semen quality: a randomized controlled study. J Endocrinol.

[41] Manna I, Jana K, Samanta PK (2004). Intensive swimming exercise-induced oxidative stress and reproductive dysfunction in male wistar rats: protective role of alpha-tocopherol succinate. Can J Appl Physiol.

[42] Khajehnasiri N, Khazali H, Sheikhzadeh F (2018). Various responses of male pituitary-gonadal axis to different intensities of long-term exercise: role of expression of KNDYrelated genes. J Biosci.

[43] Morelli A, Filippi S, Comeglio P, Sarchielli E, Cellai I, Pallecchi M (2019). Physical activity counteracts metabolic syndrome-induced hypogonadotropic hypogonadism and erectile dysfunction in the rabbit. Am J Physiol Endocrinol Metab.

